# Oral lichenoid reaction to tobacco

**DOI:** 10.11604/pamj.2016.24.330.9378

**Published:** 2016-08-30

**Authors:** Prashanth Panta, Uday Shankar Yaga

**Affiliations:** 1 Department of oral Medicine and Radiology, MNR Dental College and Hospital, Narsapur Road, Sangareddy, Telangana, India

**Keywords:** Smokeless tobacco, lichen planus, lichenoid reaction

## Image in medicine

A 55 year old male patient came for routine dental examination. History revealed that he was a heavy tobacco chewer for the past 20 years. During examination, several radiating white lines were found on top of a slightly grey background, in relation to the right buccal mucosa. The location of the lesion clearly coincided with the site of placement of quid. A differential diagnosis of lichen planus, oral lichenoid reaction and discoid lupus erythematosis were considered. On microscopic examination, there was focal perivascular infiltrate and plasma cells in the connective tissue. After correlating clinically, a diagnosis of "lichenoid reaction" was confirmed. Oral lichenoid reactions (OLR) can occur as a result of contact of an irritant such as tobacco. OLR to tobacco and betel nut products presents as a unilateral, wavy, nonelevated, non scrapable white lesion. These lines are arranged in tree like configurations or in the form of a lacy network- Wickham's striae. Wickham's striae most often are a diagnostic sign of lichen planus and lichenoid reaction.

**Figure 1:**
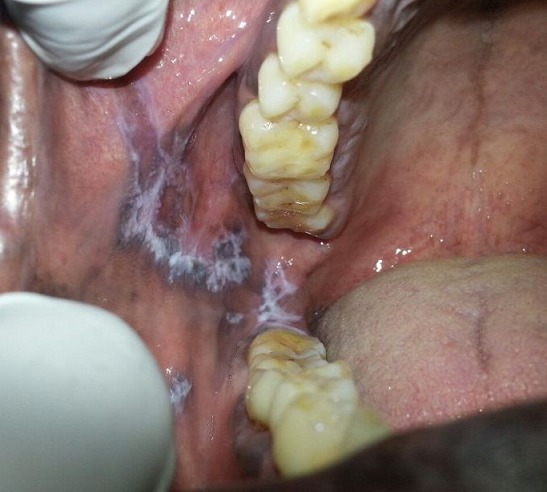
Wickham's striae

